# Feeding during the first 3 days after birth other than breast milk is associated with early cessation of exclusive breastfeeding

**DOI:** 10.1111/mcn.12971

**Published:** 2020-02-11

**Authors:** Mohammad Jyoti Raihan, Nuzhat Choudhury, Md Ahshanul Haque, Fahmida Dil Farzana, Mohammad Ali, Tahmeed Ahmed

**Affiliations:** ^1^ Nutrition and Clinical Services Division icddr,b Dhaka Bangladesh

**Keywords:** beliefs, breastfeeding initiation, breast milk, children, counselling, child feeding

## Abstract

Exclusive breastfeeding (EBF) has significant effect on morbidity and mortality. EBF is established when breastmilk alone is exclusively fed from birth until 6 months of age. However, feeding during the first 3 days after birth is often ignored for various reasons. We aimed to assess the role of feeding during the first 3 days in respect of early cessation of EBF. Data of 1,040 children aged under 6 months was derived from the baseline survey of Suchana, a large‐scale nutrition program, conducted in Sylhet, Bangladesh, and subsequently analysed. Guidelines established by World Health Organization were used to define EBF and feeding during the first 3 days. The strength of the association between feeding during the first 3 days and early cessation of EBF was established using multiple logistic regression after adjusting for other covariates. Among all children, around 62% and 13% were exclusively breastfed and were fed something other than breastmilk within the first 3 days of birth, respectively. Feeding during the first 3 days was independently and significantly associated with early cessation of breastfeeding (adjusted odds ratio: 1.94, 95% confidence interval [1.31, 2.88], *p* = .001). Less than four antenatal care (ANC) visits, increased child's age and increased household size were also independently associated with early cessation of EBF. Feeding during the first 3 days of birth is a significant predictor of early cessation of EBF. Simple counselling activities to discourage feeding anything within the first few days of birth may increase the prevalence of EBF in rural Bangladesh without investing additional resources.

**Key messages**

- Around 60% children are exclusively breastfed in rural Bangladesh.
- Feeding children anything except breastmilk during the first 3 days of birth is significantly and independently associated with the child being not exclusively breastfed later.
- Increased child's age less than the optimal number of antenatal care visits and household size are significantly associated with early cessation of breastfeeding.

## 1 INTRODUCTION

Children deprived of exclusive breastfeeding (EBF) are more likely to suffer from respiratory and other bacterial infections (Katsinde & Srinivas, 2016). Currently, around 11.6% of all under‐five mortality equating to around 800,000 child deaths are because of suboptimal breastfeeding practices, which include a deviation from EBF (World Health Organization & UNICEF, 2014). Thus, EBF and timely introduction of complementary feeding, the two critical components of optimal infant feeding practices are promoted globally to avert child mortality and morbidity (Nguyen, Withers, Hajeebhoy, & Frongillo, 2016; Pak‐Gorstein, Haq, & Graham, 2009). The Global Nutrition Targets to be achieved by 2025, is increasing the rate of EBF to, at least, 50% (World Health Organization & UNICEF, 2014) from the current global statistic of around 40% (UNICEF, 2015), whereas according to Demographic and Health Surveillance data, the current prevalence of EBF in Bangladesh is 55.3%, a decrease of 8.2 percentage points from 2011 (NIPORT, Mitra and Associates, & ICF International, 2016) despite significant stakeholders' effort to promote EBF (Ministry of Health and Family Welfare, 2016), raises concern. Reasons behind termination of breastfeeding early are multifaceted and include sociodemographic, biophysical, and psychosocial reasons. Existing evidence suggests that maternal age, father's occupation, and mode of child delivery are significantly associated with discontinuation of breastfeeding (de Oliveira & Camelo, 2017; Sun, Chen, Yin, Wu, & Gao, 2017; Venancio & Monteiro, 2006). Other aspects such as mothers' self‐efficiency, post‐natal depression, anxiety, intention to breastfeed, comfort in breastfeeding, desires or attitude of mothers regarding breastfeeding, mother–infant bonding, and family support is important in sustaining breastfeeding (Sun et al., 2017).

However, it is to be noted that EBF for children under 6 months of age as per the Demographic and Health surveys or as per World Health Organization's recommendations is established if the child did not receive anything other than breastmilk or necessary medicines in the 24 hrs preceding the survey (Greiner, 2014; NIPORT et al., 2016; World Health Organization, 2008). However, using the 24‐hr recall criteria often misclassifies children who have deviated from the criteria of EBF at some point in their life especially during the first 3 days after birth. Children delivered through caesarean section or if the child is born with low birth weight and needs special medical attention may often cause delayed initiation of breastfeeding due to delayed first mother–child contact (Dewey, Nommsen‐Rivers, Heinig, & Cohen, 2003; Rowe‐Murray & Fisher, 2002). Delayed onset of lactation may also cause children to be fed other foods until lactation normalizes, and it may take over 72 hrs or 3 days (Dewey et al., 2003). Thus, during first few days after birth, it has often been observed that many children are being shifted from EBF to predominant breastfeeding and then back to EBF (Greiner, 2014; Pak‐Gorstein et al., 2009). Also, a common phenomenon in many culture including that of Bangladesh is to provide prelacteal feed to children during the first 2 to 3 days after birth even though they are continued to be exclusively breastfed from thereafter (Ahmed, Rahman, & Alam, 1996; Greiner, 2014; Pak‐Gorstein et al., 2009; Taranum & Hyder, 1995). Nonetheless, it is also possible that easiness in feeding children other foods during early days and if it does not have any adverse effect on the child's health may discourage mothers to continue EBF. Although there is literature on a diverse range of variables explaining early cessation of EBF, there is lack of evidence clarifying the effect of feeding during the first 3 days of childbirth on early EBF cessation. This phenomenon provides the scope for this paper, which is to assess the relationship between early cessation of EBF and introduction of any feeding within the first 3 days after birth.

## 2 METHODOLOGY

The analyses are based on the baseline survey data of Suchana, a new program designed to improve the nutritional status of children and targeted the poorest segment of the population in Sylhet, Bangladesh. Cross‐sectional data of 1,040 beneficiary households including data of the youngest child aged below 6 months using a structured questionnaire was collected between November 2016 and February 2017 from the eligible Suchana beneficiaries of 80 randomly chosen unions—the lowest administrative unit of Bangladesh containing 640 vulnerable villages of Sylhet and Moulvibazar districts under Sylhet Division. The sampling procedure included the selection of 80 unions—the lowest administrative unit of Bangladesh, from a pool of 157 unions through lottery followed by a selection of vulnerable villages from the unions using participatory rural appraisal strategy. At the final stage of the selection process, systematic sampling using a calculated “interval” was used to select the households for the survey. Data were recorded digitally using personal digital assistants and maternal height, and weight was recorded using locally made wooden scales used previously in national surveys and SECA 874 model weight scale with 1‐gm resolution. Child's length was measured using SECA 416 infantometer with a precision of 1 mm.

### 2.1 Variables of interest

EBF: Infants 0–5 months of age, who received only breastmilk, and due to medical condition(s) required medicine(s) including Oral Rehydration Solution (ORS) during the previous day (World Health Organization, 2010). The variable was dichotomized into responses “yes” and “no.”

Feeding during first 3 days after birth: Whether the infant was fed anything other than the breastmilk and required medicine(s) including ORS within 3 days of birth. The variable was dichotomized into responses “yes” and “no.”

Other variables used for analyses are all pertinent to the cessation of EBF and were identified after consulting similar literature. The previous nationally representative survey questionnaires and also demographic survey questionnaire were consulted for selection of the variables relevant for the analyses (James P Grant School of Public Health [JPGSPH] & Helen Keller International [HKI], 2012; NIPORT et al., 2016). The selected variables are initiation of breastfeeding within first hour of birth, whether the child received colostrum, child's sex, child's acute morbidity status (Whether the child was sick in past 15 days before the survey), place of child delivery, maternal antenatal care (ANC) visit status, mother receiving any support on at least three household chores (cooking, gathering water/firewood for the house, cleaning, child care, agricultural activities, selling produce or going to market, homestead gardening, and homestead poultry rearing), whether mother has ever been threatened by the husband or other family members (threatened for divorce, taking another wife, and verbal or physical abuse by husband or any other family member), maternal education status, paternal education status, whether the mother received any message on breastfeeding or complementary feeding practices, maternal knowledge on EBF, maternal body mass index (BMI), household food insecurity status measured by household food insecurity access scale (Coates, Swindale, & Bilinsky, 2007), father's education status, household size, number of sleeping rooms in the household, total yearly income from all sources, maternal age, maternal age during the marriage, maternal age during the first pregnancy, maternal dietary diversity 1Measured using women dietary diversity score. (Kennedy, Ballard, & Dop, 2011), the child's age, the child's birth order, and asset score 2Asset index was constructed using principal component analysis approach using variables: ownership of cows, chickens and ducks, birds (e.g., pigeons), goats/sheep, fish, plough, unit for keeping livestock (cattle house), shop premises, unit for storing crops, boat, fishnet, rickshaw/van, trees (market retail price is above USD 1.20.), sewing machine, radio/cassette player/dvd/cd player, television, electric fan, mobile phone, bicycle, chair, table, *chouki* (cot), sofa (any type), mosquito net, ceremonial sarees, floor, roof and wall material of the house, and ownership of house(s), and the number of rooms in the household along with the type of fuel used for cooking and ownership of latrine. (NIPORT et al., 2016). Additionally, we have examined bivariate relationship between caesarean section delivery and delayed initiation of breastfeeding and also the proportion of children who were born in institutions and were fed other food or liquid during the first 3 days after birth and the proportion of children with delayed initiation of breastfeeding (>1 hr after birth) and were born in institutions.

### 2.2 Analysis procedure

All analyses were done using STATA, version 15. As the data were collected from multiple clusters, STATA command *svyset* was used for adjusting the clusters. The primary analysis involved producing descriptive statistics mentioning mean and proportion with 95% confidence interval (CI) using appropriate cut‐off values. Simple logistic regression was used to establish crude bivariate relationship between EBF and other variables. Multivariate regression was used to determine the strength of association between feeding during the first 3 days and early cessation of EBF after adjusting for necessary covariates. Variables were considered significant only if the *p* value was less than .05. Variables with a *p* value less than .25 (Bursac, Gauss, Williams, & Hosmer, 2008) were used in the final multiple logistic regression model.

### 2.3 Ethical consideration

This study was approved by the Research Review Committee and Ethical Review Committee, the two obligatory components of the institutional review board of icddr,b. Informed written consent was taken from study participants. At the beginning of each interview, the enumerator informed the respondent about the purpose of the study by reading the consent aloud in Bengali language. The respondents were also informed about their voluntary participation and their right to withdraw themselves at any point of time of the interview. While analysing the data, name or identity of the respondent was kept anonymous.

## 3 RESULTS

Of the 1,040 children assessed, 62.4% of children were exclusively breastfed and 13.0% of children had consumed anything other than breastmilk within the first 3 days of birth. All children had breastfeeding initiated within 24 hrs after birth. On other critical‐independent variables assessed, 12.8% of children did not have breastfeeding initiated within the first hour of birth, and 47.9% had acute morbidity during the last 15 days preceding the survey, whereas 83.7% of mothers had less than four ANC visits during last pregnancy and 74.0% of children were delivered at home. In total, 12% of children were delivered through caesarean section, and among all children born in institutions, 20.59% (n = 34) children had fed during the first 3 days after birth. However, for children whose breastfeeding was initiated at least 1 hr after birth, 49.62% (n = 66) were born in institutions. Additionally, 16.3% of mothers received no support from other household members for at least three household chores, and 33.9% mothers received threat from their husband. In total, 32.0% of mothers had a BMI of less than 18.5, and 20.8% mothers and 40.6% fathers had no formal education. The average age of the children was 3.77 months, whereas the average household size was 6.48 and average household yearly income from all sources was Tk. 108,809. 3USD 1 = ~Tk. 84. Details of characteristics are presented in Table 1 with results stratified by EBF status.

**Table 1:** Descriptive characteristics stratified by EBF status (*N* = 1,040)

Indicator	EBF, % [95% CI]	Non‐EBF, % [95% CI]	Overall, % [95% CI]
Whether put anything in mouth within 3 days after birth			
Yes	10.32 [6.95, 15.07]	17.39 [13.59, 21.99]	12.98 [9.99, 16.7]
Initiation breast feeding			
Within first hour of birth	87.06 [83.84, 89.71]	87.47 [83.91, 90.33]	87.21 [85.05, 89.1]
Received colostrum			
Yes	88.28 [85.57, 90.55]	84.39 [80.43, 87.68]	86.82 [84.62, 88.75]
Child's sex			
Girl	49.77 [45.59, 53.96]	48.85 [43.76, 53.96]	49.42 [45.95, 52.9]
Acute morbidity			
Yes	45.91 [42.10, 49.77]	51.15 [46.17, 56.09]	47.88 [44.85, 50.92]
At least four times visit			
At least four	19.11 [15.34, 23.53]	11.76 [8.12, 16.76]	16.35 [13.04, 20.29]
Place of delivery			
Home	74.81 [66.71, 81.49]	73.86 [70.89, 76.63]	74.03 [71.28, 76.61]
Type of delivery			
Normal	88.14 [81.42, 92.65]	87.92 [85.63, 100.0]	87.98 [85.85, 89.82]
Mother getting at least three support from household members			
Yes	85.36 [82.45, 87.86]	81.07 [76.8, 84.72]	83.75 [81.23, 85.99]
Ever threatened by husband			
No	68.10 [63.6, 72.29]	62.66 [57.71, 67.36]	66.06 [62.45, 69.49]
Mother received any message on breastfeeding or complementary feeding practices			
Yes	32.97 [28.45, 37.84]	35.81 [30.03, 42.03]	34.04 [29.84, 38.5]
Maternal knowledge of EBF			
Yes	82.74 [78.53, 86.27]	84.4 [80.21, 87.84]	83.37 [79.78, 86.42]
Maternal BMI			
Severely/moderately/mildly thin (BMI < 18.5)	30.2 [26.77, 33.86]	35.04 [29.49, 41.02]	32.02 [28.56, 35.69]
Normal (BMI: 18.5–24.99)	63.64 [59.51, 67.57]	59.34 [54.27, 64.21]	62.02 [58.51, 65.41]
Overweight (BMI: 25–30)	4.78 [3.46, 6.57]	3.84 [2.31, 6.3]	4.42 [3.32, 5.88]
Obese (BMI > 30)	1.39 [0.66, 2.87]	1.79 [0.8, 3.98]	1.54 [0.91, 2.59]
Mother's education			
No formal education	19.26 [16.39, 22.48]	23.27 [19.33, 27.74]	20.76 [18.40, 23.34]
Primary school incomplete	22.03 [19.00, 25.39]	21.73 [17.91, 26.12]	21.92 [19.50, 24.54]
Primary school completed	58.70 [54.86, 62.44]	54.98 [50.00, 59.87]	57.30 [54.27, 60.28]
Father's education			
No formal education	38.36 [34.61, 42.25]	44.50 [39.51, 49.61]	40.66 [37.64, 43.75]
Primary school incomplete	24.55 [21.32, 28.10]	20.91 [17.06, 25.35]	23.19 [20.67, 25.92]
Primary school completed	37.07 [33.36, 40.95]	34.58 [29.90, 39.57]	36.14 [33.21, 39.18]
HFIAS			
Food secure	14.95 [11.58, 19.07]	13.3 [9.52, 18.27]	14.33 [11.36, 17.91]
	EBF, mean [95% CI]	Non‐EBF, mean [95% CI]	Overall, mean [95% CI]
Child's age (months)	3.51 [3.30, 3.71]	4.20 [4.03, 4.36]	3.77 [3.59, 3.94]
Child birth order	2.98 [2.82, 3.14]	3.06 [2.83, 3.29]	3.01 [2.86, 3.16]
Maternal age (years)	25.57 [25.15, 25.98]	25.58 [25.05, 26.11]	25.57 [25.23, 25.91]
Maternal age during marriage (years)	18.25 [17.96, 18.54]	18.1 [17.85, 18.36]	18.19 [17.97, 18.41]
Maternal age during first pregnancy (years)	19.26 [18.99, 19.53]	19.07 [18.8, 19.34]	19.19 [18.98, 19.39]
Maternal WDDS	3.82 [3.70, 3.94]	3.73 [3.59, 3.87]	3.79 [3.69, 3.88]
Household size	6.37 [6.12, 6.61]	6.66 [6.4, 6.93]	6.48 [6.27, 6.68]
Number of sleeping rooms	2.02 [1.92, 2.11]	2.08 [1.98, 2.19]	2.04 [1.96, 2.12]
Total yearly income (Taka)	111,930.30 [100,542.2, 123,318.4]	103,627.90 [93,585.46, 113,670.3]	108,808.90 [100,064.4, 117,553.4]
Asset score	0.056 [−0.022, 0.136]	−0.009 [−0.104, 0.085]	0.032 [−0.029, 0.093]

Abbreviations: BMI, body mass index; CI, confidence interval; EBF, exclusive breastfeeding; HFIAS, household food insecurity access scale; WDDS, women dietary diversity score.

The simple and multiple logistic regression results suggest feeding children with anything except for breastmilk during the first 3 days after birth is both crudely and independently associated with being not exclusively breastfed later (odds ratio [OR]: 1.84, 95% CI [1.20, 2.82], *p* < .05; adjusted OR [aOR]: 1.92, 95% CI [1.31, 2.88], *p* < .05). Increased child age (aOR: 1.43, 95% CI [1.28, 1.60], *p* < .001]) less than the standard number of maternal ANC required visits (aOR: 1.58, 95% CI [1.07, 2.32], *p* < .05]) and increased household size (aOR: 1.06, 95% CI [1.01, 1.11], *p* < .05]) were also significantly associated with not being exclusively breastfed when adjusted for other variables. Our results also suggest that the odds of children who were delivered through caesarean section are around five times higher (OR: 5.28, 95% CI [3.38, 8.26], *p* < .001) to have delayed initiation of breastfeeding (after 1 hr of birth) than those children who were delivered normally. The details are provided in Table 2.

**Table 2:** Relationship between early EBF cessation and feeding during first 3 days after birth (*n* = 995)

Indicators	Unadjusted OR [CI]	*p* value	Adjusted OR [CI]	*p* value
Child was given anything within 3 days of				
No	Reference		Reference	
Yes	1.84 [1.20, 2.82]	.006	1.94 [1.31, 2.88]	.001
Initiation of breastmilk within first hour of life				
Yes	Reference		Reference	
No	0.96 [0.63, 1.46]	.861	1.00 [0.64, 1.57]	.987
Acute morbidity				
No	Reference		Reference	
Yes	1.23 [0.97, 1.57]	.088	1.10 [0.83, 1.40]	.588
Child's age	1.44 [1.30, 1.60]	<.001	1.43 [1.28, 1.60]	<.001
Standard number of ANC				
At least 4	Reference		Reference	
Less than 4	1.77 [1.21, 2.59]	.004	1.65 [1.14, 2.41]	.009
Place of delivery				
Home	Reference		Reference	
Institution	0.71 [0.52, 0.97]	.032	0.85 [0.56 1.30]	.455
Type of delivery				
Normal	Reference		Reference	
Caesarean section	1.32 [0.89, 1.96]	.162	0.97 [0.61, 1.53]	.891
Women getting support with HH tasks				
≥3 Supports	Reference		Reference	
<3 Supports	1.36 [0.99, 1.87]	.057	1.27 [0.90, 1.78]	.168
Domestic violence				
No	Reference		Reference	
Yes	1.27 [0.97, 1.66]	.077	1.09 [0.81, 1.46]	.561
Maternal BMI				
Normal (BMI: 18.5–24.99)	Reference		Reference	
Severely/moderately/mildly thin (BMI < 18.5)	1.24 [0.96.1.61]	.098	1.18 [0.88, 1.57]	.255
Overweight (BMI: 25–30)	0.86 [0.48, 1.55]	.615	1.08 [0.53, 2.20]	.826
Obese (BMI > 30)	1.38 [0.44, 4.33]	.572	1.71 [0.58, 5.03]	.325
Mother's education				
Primary school completed	Reference		Reference	
No formal education	1.29 [0.80, 1.89]	.189	1.03 [0.70, 1.52]	.863
Primary school incomplete	1.05 [0.77, 1.43]	.738	0.91 [0.66, 1.25]	.562
Father's education				
Primary school completed	Reference		Reference	
No formal education	1.24 [0.95, 1.62]	.107	1.18 [0.87, 1.60]	.268
Primary school incomplete	0.91 [0.62, 1.33]	.633	0.91 [0.60, 1.38]	.659
Household size	1.04 [1.00, 1.08]	.061	1.06 [1.01, 1.11]	.016
Yearly income	1.00 [1.00, 1.00]	.235	1.00 [1.00, 1.00]	.123

Abbreviations: ANC, antenatal care; BMI, body mass index; CI, confidence interval; EBF, exclusive breastfeeding; OR, odds ratio; HH, Household.

The post hoc analyses showed that all variables used in the multiple regression model had variance inflation factor (VIF) of less than 1.24 (mean VIF: 1.10) suggesting minimum multicollinearity. The link test *p* value of.383 suggests that there is no link error and that the link function (logit) is correctly specified. The Hosmer‐Lemeshow goodness of fit statistic suggests the model fits the data well (*p* = .484). While for model accuracy, the sensitivity was 30.1%, specificity was 84.6%, and the overall predictive accuracy was 64.2%. Finally, the ROC curve shows that the predictive capacity of the estimated model was 67.9%. Therefore, the model had a valid link function, did not have multicollinearity issue, fitted the data well, and had a reasonably good predictive capacity.

## 4 DISCUSSION

This cross‐sectional study examined the relationship between early cessation of EBF and introduction of any feeding within the first 3 days after birth in rural Bangladesh. Our findings dictate that around 60% of children were exclusively breastfed and feeding children anything except breastmilk or required medicine during the first 3 days of birth is significantly and independently associated with the child being not exclusively breastfed later. The odds of being not exclusively breastfeed is around twice for children if they had consumption of anything except breastmilk and medicines during the first 3 days after birth. Our findings also showed that the increased child's age less than the optimal number of ANC visits and household size were also significantly associated with early cessation of breastfeeding.

As to explain the occurrence of EBF cessation within the first 3 days, it could be mentioned that traditional and cultural factors play a pivotal role in introducing other feedings within first few days after birth and that may influence early discontinuation of EBF (Ahmed et al., 1996; Pak‐Gorstein et al., 2009). Anecdotal evidences of few traditional beliefs that may led to cessation of EBF are giving honey to the new born after birth, giving food so that child becomes normal and quiet, milk secretion does not happen after delivery so food is given, and mustard oil is given to clear the child's oral cavity (Taranum & Hyder, 1995). Infant feeding practices in communities and maternal decision to breastfed are strongly influenced by people's perception, knowledge, and beliefs about everyday life (Ahmed et al., 1996; Pak‐Gorstein et al., 2009; Roy, Dasgupta, & Pal, 2009). Cultural aspects regarding feeding foods and liquids other than breastmilk should also be taken into consideration when studying early introduction of other foods (Ahmed et al., 1996; Lakati, Makokha, Binns, & Kombe, 2011; Pak‐Gorstein et al., 2009; Taranum & Hyder, 1995). Additionally, complications arising post‐partum such as maternal inability to breastfeed due to the consumption of certain medications and breastfeeding failure due to incorrect positioning of the child may also influence mothers to initiate feeding of other foods or cease breastfeeding promptly after giving birth (Perera, Ranathunga, Fernando, Sampath, & Samaranayake, 2012). Nonetheless, as indicated by our result, simply by decreasing the proportion of children not being fed anything except for breastmilk and required medicine during the first 3 days of life would prompt for further increase in the prevalence of EBF without investing extra resources. Therefore, implementing effective communication strategies for behavioural change and attitudes towards introducing any other feeds within the first 3 days after birth at rural community level should be of great importance. Open discussion sessions with mothers about breastfeeding issues to build confidence can be incorporated in programs for promoting EBF discussion on this issue (Ku & Chow, 2010). Discussions and other EBF promotional activities can be inclusive of friends, other senior members of the family, and in‐laws to improve the uptake of the concept of EBF (Chandrashekhar et al., 2007; Khanal, Adhikari, Sauer, & Zhao, 2013).

On other covariates examined, increased age of the child was also found to be significantly associated with cessation of EBF in our study. Other studies also reported similar findings (Perera et al., 2012; Victor, Baines, Agho, & Dibley, 2013). As per a report published on an initiative to improve infant feeding practices in Bangladesh also showed the rate of EBF to decline sharply with increased child's age (Saha et al., 2011). A cohort study from neighbouring Sri Lanka indicated that maternal anxiety over inadequate breastmilk production and not achieving optimal weight gain for age to be the most important reasons for early EBF cessation (Perera et al., 2012). In our study, less than four ANC visits was found to be significantly associated with early cessation of EBF. It is possible that the mothers who attended the standard number of four ANCs have retained the counselling they received on EBF and acted accordingly (Nekatebeb, Guyon, Beyero, & Stoecker, 2010). Stated by a previously conducted study, ANC visit is an important access point that can improve maternal breastfeeding behaviour (Tariku et al., 2017) and antenatal feeding intention was significantly associated with early breastfeeding cessation (Oakley, Henderson, Redshaw, & Quigley, 2014).

On the significance of increased household size being associated with early cessation of EBF, it could be said that the reasons are multifaceted; increased household size reflects narrow interval between births, and mothers may have to cease EBF as a preparation for pregnancy (Akter & Rahman, 2010a; Akter & Rahman, 2010b; Reddy & Abuka, 2016). Additionally, other factors pertaining to higher household size and parity such as previous adverse breastfeeding experience (de Oliveira & Camelo, 2017) and poor health of mother due to high parity (Giashuddin, Kabir, Rahman, & Hannan, 2003) may cause early cessation of EBF. Elderlies in the family especially grandmothers play a critical role in support and care‐seeking of children (Saha et al., 2011). A study in Bangladesh has shown that in around 42% of EBF cessation cases, food was introduced by the grandmother, followed by the traditional birth attendant (24.5%) (Taranum & Hyder, 1995).

It is evident that breastfeeding is the ideal nourishment for a child up to 6 months of age. It remained crucial to achieving fourth millennium development goals and a priority in the second sustainable development goal (Kaur, 2017). The scaling up of breastfeeding can prevent an estimated 82 3000 child death each year. Breastfeeding promotion is important in both developing and developed countries alike and might contribute to the achievement of forthcoming sustainable development goals (Victora et al., 2016).

Considering our findings, it is recommended to promote EBF for the first 3 days of life through intensifying existing programs under National Nutrition Services (DGHS, 2019) as it could significantly increase the prevalence of EBF without involving much resources and could be a critical intervention for child's health in resource‐poor settings. Our study further reiterates the importance of ANC visits in promoting ideal breastfeeding practices among expectant mothers and the community, especially the family, taking into account the local traditions and customs. Another key element to promote breastfeeding is to create a more enabling environment for effective behaviour change communications between the care givers and seekers as breastfeeding is both a natural and a learned behaviour (World Health Organization, 2003). The objective can be achieved by increasing the number of trained facilitators and by enhancing the skills of the health care providers involved in promoting EBF (Giashuddin et al., 2003; Saha et al., 2011) and also by ensuring more focused monitoring of the pertaining activities by the stakeholders.

### 4.1 Limitations and strengths

For the purpose of collecting data on infant and young child feeding, we had to depend solely on the respondents' response. There is a probability that some of the responses are prone to recall bias; however, the data collectors were specifically trained to collect those responses as accurately as possible. The cross‐sectional nature of the study prevents it from drawing any causal inference; however, a large sample size was added to the strength of the study.

## CONFLICTS OF INTEREST

The authors declare that they have no conflicts of interest.

## CONTRIBUTIONS

NC conceptualized the manuscript. MA, MAH, FDF, and MJR have performed statistical analysis. NC, MA, FDF, and MJR drafted the manuscript. TA and NC reviewed and edited the manuscript. All authors have contributed to the revision of the final draft before submission. All authors have read and approved the final version and are responsible for the final content of this manuscript.
